# Use of BERT (Bidirectional Encoder Representations from Transformers)-Based Deep Learning Method for Extracting Evidences in Chinese Radiology Reports: Development of a Computer-Aided Liver Cancer Diagnosis Framework

**DOI:** 10.2196/19689

**Published:** 2021-01-12

**Authors:** Honglei Liu, Zhiqiang Zhang, Yan Xu, Ni Wang, Yanqun Huang, Zhenghan Yang, Rui Jiang, Hui Chen

**Affiliations:** 1 School of Biomedical Engineering, Capital Medical University Beijing China; 2 Beijing Key Laboratory of Fundamental Research on Biomechanics in Clinical Application, Capital Medical University Beijing China; 3 Department of Radiology, Beijing Friendship Hospital, Capital Medical University Beijing China; 4 Ministry of Education Key Laboratory of Bioinformatics, Research Department of Bioinformatics at the Beijing National Research Center for Information Science and Technology, Center for Synthetic and Systems Biology Department of Automation Tsinghua University Beijing China

**Keywords:** BiLSTM-CRF, natural language processing, radiology reports, information extraction, computer-aided diagnosis, BERT

## Abstract

**Background:**

Liver cancer is a substantial disease burden in China. As one of the primary diagnostic tools for detecting liver cancer, dynamic contrast-enhanced computed tomography provides detailed evidences for diagnosis that are recorded in free-text radiology reports.

**Objective:**

The aim of our study was to apply a deep learning model and rule-based natural language processing (NLP) method to identify evidences for liver cancer diagnosis automatically.

**Methods:**

We proposed a pretrained, fine-tuned BERT (Bidirectional Encoder Representations from Transformers)-based BiLSTM-CRF (Bidirectional Long Short-Term Memory-Conditional Random Field) model to recognize the phrases of APHE (hyperintense enhancement in the arterial phase) and PDPH (hypointense in the portal and delayed phases). To identify more essential diagnostic evidences, we used the traditional rule-based NLP methods for the extraction of radiological features. APHE, PDPH, and other extracted radiological features were used to design a computer-aided liver cancer diagnosis framework by random forest.

**Results:**

The BERT-BiLSTM-CRF predicted the phrases of APHE and PDPH with an F1 score of 98.40% and 90.67%, respectively. The prediction model using combined features had a higher performance (F1 score, 88.55%) than those using APHE and PDPH (84.88%) or other extracted radiological features (83.52%). APHE and PDPH were the top 2 essential features for liver cancer diagnosis.

**Conclusions:**

This work was a comprehensive NLP study, wherein we identified evidences for the diagnosis of liver cancer from Chinese radiology reports, considering both clinical knowledge and radiology findings. The BERT-based deep learning method for the extraction of diagnostic evidence achieved state-of-the-art performance. The high performance proves the feasibility of the BERT-BiLSTM-CRF model in information extraction from Chinese radiology reports. The findings of our study suggest that the deep learning–based method for automatically identifying evidences for diagnosis can be extended to other types of Chinese clinical texts.

## Introduction

In the past decades, electronic health records (EHRs) from millions of patients have become massive sources of valuable clinical data. Machine learning–based algorithms, especially deep learning algorithms, have been applied effectively to analyze patient data and they have shown promising results, thereby advancing medical research and better informing clinical decision making for the secondary use of EHRs [1,2]. Owing to the high dimensionality, noise, heterogeneity, random errors, and systematic biases, the data mining of EHRs remains challenging. Natural language processing (NLP) technologies could extract meaningful information, thus facilitating the application of clinical texts. There are 2 types of methods for information extraction, namely, rule-based methods and machine learning methods [1]. The use of machine learning methods for data mining of EHRs can derive previously unknown clinical insights and be applied powerfully in clinical decision-making and computer-aided diagnosis of diseases [3,4]. Recently, deep learning methods have had a profound impact in various areas of research because of their simplicity, efficient processing, and state-of-the-art results [5,6]. In particular, recurrent neural networks and Word2Vec embedding are the most popular methods that are utilized in clinical NLP tasks [2]. Deep learning methods have made improvements in various clinical applications, especially for text classification, named-entity recognition (NER), relation extraction, and question answering [7,8]. With growing acceptance and increasing number of applications, deep learning methods have become a baseline in many clinical NLP tasks.

Word embedding is an essential step for sequencing labelling tasks. Learning word representations from massive unannotated documents is a long-established method. The Word2Vec method [9] is the first word embedding approach based on deep learning methods. The model derives the semantic and synthetic meaning of a word on account of its adjacent words by using unsupervised learning. Global Vector word representation [10] is another effective word embedding method, which constructs a global word-word co-occurrence matrix and utilizes matrix factorization to learn embeddings in a lower dimensional space. However, the word-level representations have a limitation that only a single embedding is provided for a word with no thought for polysemy under different contexts. Unlike the traditional embedding methods, ELMo (Embeddings from Language Models) [11] uses a bidirectional language model to embed the context information into word representations. BERT (Bidirectional Encoder Representations from Transformers) [12] is another prominent contextualized word representation model, which uses a masked language model that predicts randomly masked words in a context sequence. Different from ELMo, BERT targets different training objectives and uses a masked language model to learn bidirectional representations. For clinical sequence labelling tasks such as NER, rule-based approach and conditional random fields (CRFs) have been used widely. Deep learning technologies substantially improve the NER performance through multi-layer data representations. Of the popular deep learning methods, BiLSTM (bidirectional long short-term memory) can capture long-range related information effectively. Furthermore, BiLSTM with CRF, known as BiLSTM-CRF, outperforms the traditional models with feature extraction and reduces the workload of feature selection [13].

Due to the difference in the grammatical features from English and the limitation of the EHR corpus, information extraction of Chinese EHRs using NLP remains challenging. In the medical field, researchers have developed information extraction algorithms for varied implementations, including diagnostic models for different diseases such as cancers [14] and childhood diseases [15]. For Chinese NER tasks, BiLSTM-CRF is the most common and practical approach [16,17]. BERT has also received extensive attention in Chinese EHRs. Zhang et al used fine-tuning BERT for NER and relation extraction in several types of Chinese clinical documents. The comprehensive clinical information related to breast cancer was extracted [14]. Wu et al developed an aided clinical diagnosis service on EHRs by using a deep learning model [3]. Liang et al applied an automatic NLP system and achieved a high diagnostic accuracy in childhood diseases [15].

The radiology report is a crucial component of EHRs, as it is the communication bridge between radiologists and physicians. The accuracy and efficiency of diagnosis are limited since it is formulated based on subjective judgment, especially for inexperienced physicians. Hence, extracting useful radiological information from radiology reports has considerable significance in advancing radiological research and clinical practice [18,19]. NLP technologies have received great attention in the processing of radiology reports and have been successfully applied in identifying biomedical concepts [20], extracting recommendations [21], determining the change level of clinical findings [22], and so on.

With the development of machine learning methods in recent eras, computer-aided early diagnosis for cancer based on massive clinical data becomes feasible. Many diseases have been investigated to date, such as hepatocellular cancer [23] and colorectal cancer [24]. In this study, we focused on the computer-aided diagnosis of liver cancer, which remains to be a substantial disease burden in China. For liver cancer diagnosis, dynamic contrast-enhanced computed tomography (CT) is one of the primary diagnostic tests. Imaging findings of the key enhanced scan phases such as the arterial phase, portal phase, and delayed phase are recorded in the radiology reports. According to the guidelines of the Chinese Society of Clinical Oncology (CSCO), hyperintense enhancement in the arterial phase (APHE) and hypointense enhancement in the portal and delayed phases (PDPH) are significant diagnostic evidences for liver cancer [25].

In this study, we designed deep learning–based methods to identify evidences for liver cancer diagnosis automatically. We recognized the phrases of APHE and PDPH by using a BERT-BiLSTM-CRF model by combining a pretrained, fine-tuned BERT language model with BiLSTM-CRF. We also applied the FENLP (feature extraction using the rule-based NLP) method based on the content of radiology reports to extract the radiological features. Therefore, the evidences for diagnosis, considering both clinical knowledge and radiology findings, contained APHE, PDPH, and radiological features extracted by FENLP [26]. With these evidences, we designed a computer-aided liver cancer diagnosis framework by using random forest.

## Methods

### Evidence Extraction for Diagnosis of Liver Cancer

**Figure 1:**
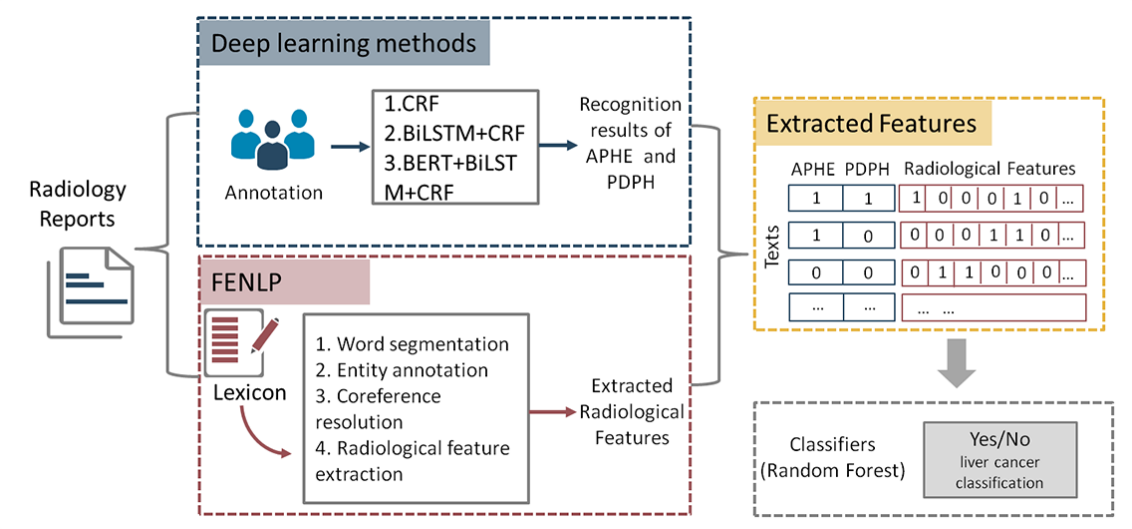
The workflow of this research. Labels 0/1 represent the absence/presence of a certain feature. BERT: Bidirectional Encoder Representations from Transformers; BiLSTM: bidirectional long short-term memory; CRF: conditional random field; APHE: hyperintense enhancement in the arterial phase; PDPH: hypointense in the portal and delayed phases.

Figure 1 shows the workflow of the evidence extraction for the diagnosis of liver cancer. We implemented 2 feature extraction methods based on clinical knowledge and the content of radiology reports to generate a radiological feature set. Then, we built a random forest model to predict liver cancer by using these features as inputs.

### Data Sets

We collected abdomen and pelvic CT radiology reports from a tertiary hospital in Beijing, China, between 2012 and 2019. To protect patient privacy, we removed all the identifying information. An unstructured radiology report has different sections, including Type of Examination, Clinical History, Comparison, Technique, Imaging Findings, and Impressions. The Impressions section summarizes crucial radiology findings from the Findings section and contains a diagnosis indicated by a radiologist. In this study, the diagnosis of liver cancer was determined according to the Impression section and the annotation of experienced radiologists, resulting in 480 patients with liver cancer. We randomly selected 609 patients without liver cancer from our data set. Therefore, 480 and 609 radiology reports for patients with and without liver cancer, respectively, were used in this study. We then trained and evaluated an NER model on the Imaging Findings section. The reports were randomly divided into the training set and the test set in a ratio of 8:2.

### BERT-BiLSTM-CRF for Recognition of APHE and PDPH

We considered the recognition of APHE and PDPH as a sequence labelling task at the character level, where the goal was to assign the BIO (Begin, Inside, Outside) tags to each Chinese character. In this study, BIO tags contained B-APHE, I-APHE, B-PDPH, I-PDPH, and O-Outside. We invited 2 radiologists with more than 5 years of medical experience to annotate all the data. If there was any inconsistency, another experienced radiological expert was then asked to make the final annotation, to obtain the gold standard annotated data. To ensure the consistency of the annotation, radiologists were trained in advance. At the report level, APHE and PDPH were not mutually exclusive, that is, 1 report could contain both APHE and PDPH. Of all the reports, 602 had the phrase of APHE and 330 had the phrase of PDPH. For the 480 reports diagnosed with liver cancer, the numbers of APHE and PDPH were 442 and 330, respectively.

**Figure 2:**
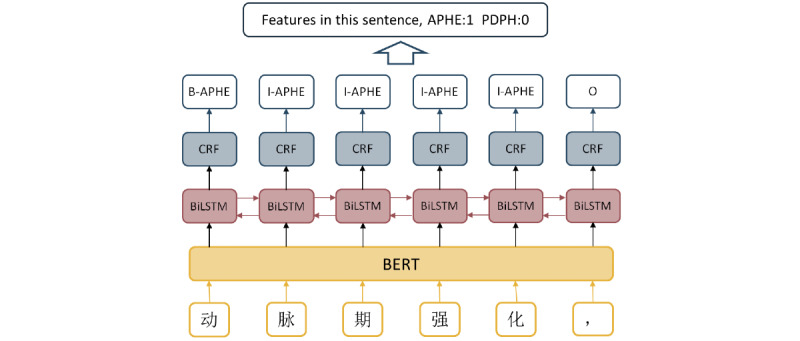
The architecture of the BERT-BiLSTM-CRF model for the recognition of APHE and PDPH. BERT: Bidirectional Encoder Representations from Transformers; BiLSTM: bidirectional long short-term memory; CRF: conditional random field; APHE: hyperintense enhancement in the arterial phase; PDPH: hypointense in the portal and delayed phases.

BiLSTM-CRF is commonly used in the sequence labeling task. To further improve the recognition performance for the features of APHE and PDPH, we performed the BERT-BiLSTM-CRF model comprising a fine-tuned BERT language model for word embedding and BiLSTM-CRF method for feature recognition. CRF and BiLSTM-CRF model were applied as the baseline. APHE and PDPH in Chinese radiology reports had a variety of presentations such as detailed presentation, CT values of different phases, and abbreviations (Table 1). The deep learning model for the recognition of APHE and PDPH consisted of 3 layers, namely, the word embedding layer, BiLSTM layer, and CRF layer (Figure 2).

**Table 1:** Some expressions of APHE^a^ and PDPH^b^ in Chinese.

Expressions of APHE and PDPH in Chinese	Detailed descriptions
增强后动脉期明显不均匀强化	The arterial phase shows the heterogeneous density in the enhanced scan.
动脉期强化明显	Marked enhancement is shown in the arterial phase.
动脉期可见多发强化灶	Multiple enhancement areas are seen in the arterial phase.
门脉期相对低密度	The portal phase has relatively low density.
门脉期可见消退	PDPH occurs in the portal phase.

^a^APHE: hyperintense enhancement in the arterial phase.

^b^PDPH: hypointense in the portal and delayed phases.

#### Word Embedding Layer

The word embedding layer could map and transform the discrete characters into distributed representations. A word-level vector representation learned a real valued vector to represent a word from a large amount of unannotated text. On most NLP tasks, BERT could achieve state-of-the-art performance while requiring minimal architectural modification [27]. In this study, we applied Word2Vec and BERT to train the character vectors, followed by a comparison of the results. The Word2Vec was used with a dimension size of 100 and a batch size of 120. The Word2Vec was pretrained on the Chinese Wikipedia data. The sentence embedding had been pretrained and fine-tuned by BERT on the original Google BERT GitHub repository [28]. The maximum sequence length was set to 256 with a batch size of 64.

#### BiLSTM Layer

Recurrent neural networks is a family of neural networks, which is usually used for modelling sequential data. The LSTM (Long Short-Term Memory Networks) is a variant of the recurrent neural networks, and it can effectively capture high dependencies and retrieve rich global information. LSTM solves the problem by using the gating mechanism. An LSTM unit consists of 3 gates (ie, Input Gate, Output Gate, and Forget Gate), which can select semantic information in a neural network. Compared with LSTM, BiLSTM can learn forward and backward information of input words by splitting the neurons into 2 directions of a text sequence. We set the number of hidden units in BiLSTM to 100 and the optimizer to Adam.

#### CRF Layer

For the sequence labelling step in our study, adjacent tags had dependencies. For example, an inside tag I must follow a begin tag B. We applied the sequential CRF to calculate optimal sequence combinations on top of the BiLSTM layer that could consider the dependencies of adjacent tags.

#### APHE and PDPH Labels at the Report Level

Considering the characteristics of Chinese language and also avoiding the noise, we defined the following as APHE or PDPH features at the report level: (1) 2 continuous characters that were the abbreviations of APHE (ie, 快进) or PDPH (ie, 快出); (2) more than 3 continuous characters that were predicted as APHE or PDPH. Criterion (1) was checked first and was only based on the characters. If not met, criterion (2) was checked, which was based on CRF results.

#### FENLP for Radiological Feature Extraction

We implemented the NLP pipeline in the Findings section to extract useful features from the unstructured radiology reports to facilitate liver cancer diagnosis. As shown in Figure 1, the NLP pipeline consisted of 4 successive steps, that is, word segmentation, entity annotation, coreference resolution, and relationship extraction, resulting in radiological features consisting of 1 or more terms. The detailed description of the pipeline is provided in our previous study [26]. The whole pipeline was based on a lexicon that was constructed manually according to Chinese grammatical characteristics. A small number of reports were sampled randomly for generating the lexicon by manual reading. The lexicon contained clinical terms and lists of synonyms. The lexicon was collected in the same hospital and clinical text type with this study. Five entity types (Location, Morphology, Density, Enhancement, and Modifier) were recognized. After coreference resolution, according to the synonym list in the lexicon, we then used several patterns of entity types as rules to obtain the final radiological features (Table S1 of Multimedia Appendix 1). Therefore, the radiological features could be seen as a combination of several entities such as 肝脏+低密度影 (liver + low density) and 肝脏+增强扫描未见强化 (liver + enhancement scan showed no enhancement).

#### Prediction Models

Using the radiological features obtained by BERT-BILSTM-CRF and FENLP, we used a random forest model for the liver cancer diagnosis. Random forest is an ensemble learning method constructed with a multitude of decision trees, which is widely used in classification tasks. The performance was measured by the recall, precision, and F1 score. Random forest could generate the feature importance score, which was computed by Gini impurity. Gini impurity is a measurement of the probability that a sample is classified incorrectly without a specific feature. In our study, the higher the feature importance score of the radiological features was, the more linked it was with the liver cancer diagnosis. We used the feature importance score to rank all the radiological features.

## Results

We extracted the features of APHE and PDPH by using 3 different models, that is, CRF, BiLSTM-CRF, and BERT-BiLSTM-CRF. The recognition results were presented both at the report level and character level (Table 2). At the report level, the performance was computed depending on whether the radiology reports contained a feature of APHE or PDPH. At the character level, the recognition results of BIO tags for each Chinese character were counted. For the character-level recognition results of APHE and PDPH, the BERT-BiLSTM-CRF model obtained the best performance, with F1 scores of 89.14% and 82.19%, respectively. At the report level, the BERT-BiLSTM-CRF model also achieved the best performance (F1 scores of 98.40% for APHE and 90.67% for PDPH). For the other 2 baseline models, the BiLSTM-CRF model outperformed the CRF model but underperformed the BERT-BiLSTM-CRF model. If a single character was recognized as a feature, it would be regarded as noisy information, thereby leading to its exclusion from the report-level results. As a result, the recognition performances at the report level were higher than those at the character level in all the models. We chose the recognition results of APHE and PDPH at the report level by the BERT-BiLSTM-CRF model as the predictors for further liver cancer diagnosis.

The feature extraction method FENLP used the lexicon described in our previous study, which included 831 words and 5 entity types. Entity combinations conforming to specific entity patterns were formulated as radiological features. The patterns included Location + Density, Location + Enhancement, Location + Enhancement + Modifier, Location + Density + Modifier, and Location + Morphology. We retained the radiological features that occurred more than twice. We finally obtained 301 radiological features; among them, 6 features had a frequency higher than 300 (Table S2 of Multimedia Appendix 1).

**Table 2:** Recognition performance of APHE^a^ and PDPH^b^ by using different models at the character level and report level.

Models			Accuracy (%)	Precision (%)	Recall (%)	F1 score (%)
**Character level**						
	**Conditional random field**					
		APHE	96.05	84.13	72.19	77.70
		PDPH	97.44	80.37	59.02	68.06
	**Bidirectional long short-term memory-conditional random field**					
		APHE	97.54	90.86	82.56	86.51
		PDPH	98.24	84.56	75.72	79.89
	**BERT^c^+ Bidirectional long short-term memory-conditional random field**					
		APHE	97.97	91.14	87.22	89.14
		PDPH	98.46	88.60	76.64	82.19
**Report level**						
	**Conditional random field**					
		APHE	94.52	98.28	91.94	95.00
		PDPH	89.00	87.69	79.17	83.21
	**Bidirectional long short-term memory-conditional random field**					
		APHE	95.89	97.30	94.74	96.00
		PDPH	93.61	92.19	86.76	89.39
	**BERT+** **Bidirectional long short-term memory-conditional random field**					
		APHE	98.17	97.62	99.19	98.40
		PDPH	93.61	87.18	94.44	90.67

^a^APHE: hyperintense enhancement in the arterial phase.

^b^PDPH: hypointense in the portal and delayed phases.

^c^BERT: Bidirectional Encoder Representations from Transformers.

**Figure 3:**
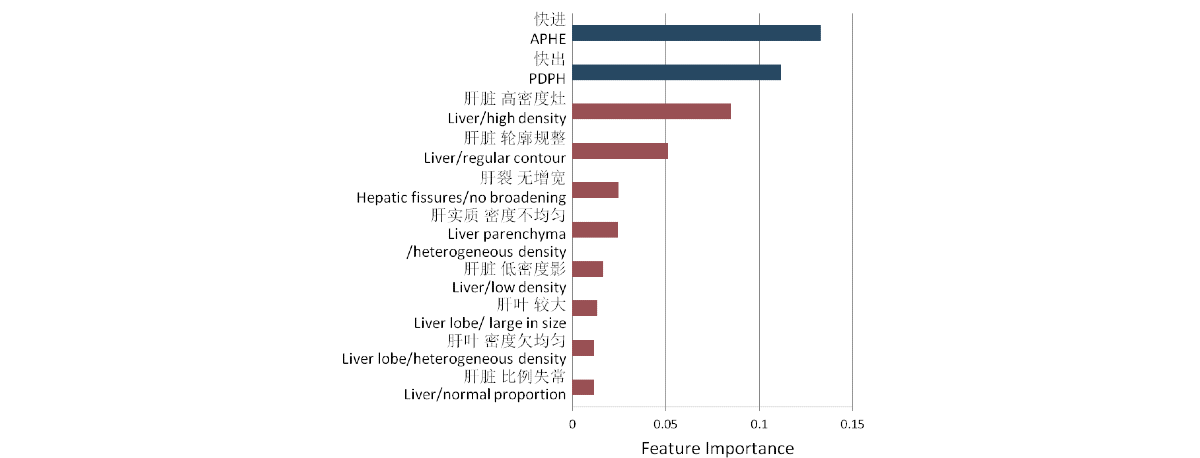
Top 10 radiological features linked with liver cancer diagnosis ranked by feature importance score. APHE: hyperintense enhancement in the arterial phase; PDPH: hypointense in the portal and delayed phases.

According to the presence or absence of each feature extracted from either BERT-BILSTM-CRF or FENLP, each radiology report was represented by a 0-1 vector. The prediction results using different patterns of features are shown in Table 3. F1 scores of random forest using features from BERT-BILSTM-CRF and FENLP were 84.88% and 83.92%, respectively. With a combination of both kinds of features, the final F1 score of prediction model increased to 88.55%. Among all the feature input patterns, the precision and accuracy also obtained the highest value while inputting all the features, while the prediction model had the highest recall rate with only 2 features of APHE and PDPH. Among the features with a frequency higher than 10 in all the reports, the top 10 features linked with the liver cancer diagnosis were identified with the feature importance score computed by Gini impurity (Figure 3). The top 2 features were APHE and PDPH, which had substantially larger feature importance scores than the other features extracted from FENLP.

**Table 3:** Performance of different patterns of features for liver cancer diagnosis.

Patterns of Features	Accuracy (%)	Precision (%)	Recall (%)	F1 score (%)
APHE^a^ and PDPH^b^	86.11	81.38	88.70	84.88
Radiological features from FENLP^c^	85.71	83.06	84.80	83.92
All features	90.25	91.52	85.77	88.55

^a^APHE: hyperintense enhancement in the arterial phase.

^b^PDPH: hypointense in the portal and delayed phases.

^c^FENLP: feature extraction using the rule-based natural language processing.

## Discussion

### Principal Results

Diagnostic prediction of cancer by using data mining methods is an essential and significant application of EHRs [5]. From previous studies, features extracted from EHRs have proved to be the valid input of the diagnostic model [14,29]. In particular, the use of machine learning methods, especially deep learning methods for clinical information extraction, could facilitate in providing new evidences in computer-aided diagnosis. As the burden of liver cancer is widely accepted as one of the principal and universal challenges in health care and as patients with liver cancer are usually diagnosed at the terminal stage, the early and accurate diagnosis of liver cancer by radiology examination has great significance [30,31]. In contrast with previous studies of liver cancer diagnosis, our study focused on the identification of evidences for live cancer diagnosis from Chinese radiology reports. We selected APHE and PDPH as the known evidences for diagnosis according to the guidelines of CSCO. These 2 features were essential but not sufficient enough to represent the whole report and further be used to diagnose liver cancer. Furthermore, using FENLP, we also extracted uncertain numbers of radiological features from the report content, because we aimed to obtain new evidences for essential diagnosis. Therefore, the evidences for diagnosis were obtained both from clinical knowledge and the content of reports. For the recognition of APHE and PDPH, we applied BERT on word embedding in the deep learning method, which achieved state-of-the-art performance.

Word embedding is an essential step for sequencing labelling tasks. Previously popular models such as Word2Vec and Global Vector word representation focused on learning context-independent word representations. Recent advances in word representations based on language models, including ELMo, CoVe, and BERT, could dynamically improve the word representations and discriminate among multiple meanings of a word. In particular, based on the attention mechanism, BERT exhibited an upward trend and outperformed the previous models in many NLP tasks. Recognition of APHE and PDPH using traditional NLP methods had difficulties, because the related descriptions covered varied Chinese sentence structures and entity types (Table 1). For example, for hyperintense enhancement, the sentence pattern and phrase could have different styles due to the different writing habits of different radiologists or due to the use of Chinese abbreviations. Different from the Word2Vec model, BERT learned context-dependent word representations by using bidirectional transformers. The BiLSTM algorithms are widely used and easily implanted in sequence-related work such as entity extraction. We annotated all the characters in the Findings section manually with BIO tags and then applied the BERT-BiLSTM-CRF model to recognize APHE and PDPH. The high performance proved the feasibility of the BERT-BiLSTM-CRF model in information extraction from Chinese radiology reports.

In this study, among the recognition results of APHE and PDPH obtained by the 3 different models, the BERT-BILSTM-CRF model finally achieved the best performance for both APHE (F1 score 98.40%, precision 97.62%, and recall 99.19%) and PDPH (90.67%, 87.18%, and 94.44%, respectively) at the report level. For the 2 baseline models based on CRF, the model with a BiLSTM layer received a much higher F1 score than the model without a BiLSTM layer. The results indicated that, with the word embedding language model BERT and the BiLSTM model, the recognition of APHE and PDPH could result in much higher performance. To avoid the noise in the recognition results, we used the recognition results at the report level to be the input radiological features of the liver cancer diagnostic model. Report-level recognition concerned only continuous characters longer than 3 characters and specific Chinese abbreviations. Therefore, report-level results could represent whether the report contained the features of APHE or PDPH. The recognition of APHE and PDPH by BERT-BiLSTM-CRF was accurate enough to be the predictors of liver cancer diagnosis.

Only 2 fixed features of APHE and PDPH were not enough for liver cancer diagnosis. Therefore, we further performed the automatic NLP pipeline FENLP to extend the feature set based on Chinese grammar and radiological characteristics. Different from that of BERT-BILSTM-CRF, the number of features generated by FENLP was unknown and changed according to the training texts. In this study, we finally extracted 301 features. The top features were the typical morphology of the different locations, which were essential to the diagnosis of the liver diseases (Table S2 of Multimedia Appendix 1).

We chose the random forest as the liver cancer diagnostic model. With 2 kinds of features obtained by BERT-BILSTM-CRF and FENLP, random forest could reach an F1 score of 88.55%, which was much higher than the model using either kind of features. The performance of the diagnostic model using APHE and PDPH was slightly higher than that of the model using features extracted from FENLP. By contrast, FENLP produced much more features than BERT-BILSTM-CRF. We further ranked the features by the feature importance score computed by Gini impurity, which could reflect the degree of association with liver cancer. APHE and PDPH were the top 2 features with a clearly higher feature importance score compared with other features obtained by FENLP. The results indicated the strong association of APHE and PDPH with liver cancer, which coincided with the current clinical knowledge. Of the top features obtained by FENLP, the feature *high density of liver* had the highest feature importance score, which was the important and basic risk factor for the diagnosis of liver diseases. Broadening of hepatic fissures was an essential feature that existed in liver cirrhosis or in liver cancer progressed from liver cirrhosis [30]. Our results confirmed that the radiological features from FENLP could also be an evidence for diagnosis, which could further improve the diagnostic performance. Furthermore, the top features linked with liver cancer could extend the diagnostic evidence and be the supplementary features of APHE and PDPH.

Designing disease diagnostic models based on EHRs is a significantly important research field. Recently, NLP and deep learning-based models have been widely applied in many studies [7]. For instance, Sada et al designed and performed NLP-assisted radiology document classification for liver cancer detection. The model finally received an F1 score of 0.78 [23]. Compared with previous studies on clinical information extraction, the evidences for diagnosis in this study were identified based on the clinical knowledge from the guidelines of CSCO and the content of the reports. APHE and PDPH are 2 widely accepted evidences for disease diagnosis, and they have also proved to be essential features in our liver cancer diagnostic model. Other radiological features from FENLP enlarged the potential evidences for the diagnosis of liver cancer. Moreover, we utilized the BERT-BiLSTM-CRF model in this study, which achieved the state-of-the-art performance.

### Limitations

Our study had the following limitations. The number of radiological features from FENLP was not fixed since all desirable features were retained, which might introduce some noise into the extracted radiological features. Besides, from the clinical knowledge in the guidelines of CSCO, we only extracted 2 characteristic features. In future, we will collect more evidences for diagnosis in order to further improve the performance and make the model more explanatory. Through the analysis of the misjudged samples in the recognition of APHE and PDPH, we identified the main error that occurred when the description of APHE and PDPH only included CT values. With the comparison of CT values in different phases, we could define these 2 features. However, our methods did not focus on the CT value extraction, and the number of these cases were small. In future studies, CT value extraction and analysis can avoid this kind of error and increase the prediction performance.

### Conclusion

In this study, we developed a deep learning–based method for the recognition of evidences for disease diagnosis and designed a computer-aided liver cancer diagnosis framework. The diagnostic evidences contained APHE, PDPH, and radiological features extracted by FENLP. We proposed the BERT-based deep learning model BERT-BILSTM-CRF for recognizing the phrases of APHE and PDPH, which are the essential features associated with liver cancer diagnosis. Our work confirms that BERT-based deep learning model can be used and has desirable performance in the radiological feature extraction of Chinese radiology reports. Furthermore, this study was a comprehensive study for NLP and its application, focusing on Chinese radiology reports. The deep learning model proposed in this study for information extraction is expected to be extended to different types of Chinese clinical texts and other kinds of applications.

## Appendix

Multimedia Appendix 1 Supplementary data.

## Abbreviations

APHE: hyperintense enhancement in the arterial phase
BERT: Bidirectional Encoder Representations from Transformers
BiLSTM: bidirectional long short-term memory
BIO: Begin, Inside, Outside
CRF: conditional random field
CSCO: Chinese Society of Clinical Oncology
CT: computed tomography
EHR: electronic health record
ELMo: Embeddings from Language Model
FENLP: feature extraction using the rule-based natural language processing
NER: named-entity recognition
NLP: natural language processing
PDPH: hypointense in the portal and delayed phases
