# Supplementary material for: Use of BERT (Bidirectional Encoder Representations from Transformers)-Based Deep Learning Method for Extracting Evidences in Chinese Radiology Reports: Development of a Computer-Aided Liver Cancer Diagnosis Framework
Source: J Med Internet Res. 2021 Jan 12;23(1):e19689. doi: 10.2196/19689 (PMC7837998; doi:10.2196/19689)
Supplement: Multimedia Appendix 1 [file jmir_v23i1e19689_app1.docx]

**Supplementary Files**

Table S1. Patterns summarized to extract features according to the word’s entity type.

| Entity pattern | Example |
| --- | --- |
| Location + Density | 肝脏+低密度影 (liver + low density)  肝脏+高密度影 (liver + high density) |
| Location + Enhancement | 肝脏+增强扫描未见强化  (liver + enhancement scan showed no enhancement) |
| Location + Enhancement + Modifier | 肝门+动脉期+结节状强化(porta hepatis + arterial phase + nodular enhancement) |
| Location + Density + Modifier | 肝脏+低密度灶+边界清晰(liver + low density area + clear boundary) |
| Location + Morphology | 肝脏+形态大小正常 (liver + normal in size and shape)  肝脏+轮廓规整 (liver + normal contour) |

Table S2. Radiological features with a frequency greater than 300 obtained by FENLP.

| Radiological Features | Count | Proportion in all the reports | Proportion in all the features |
| --- | --- | --- | --- |
| 肝脏 / 形态大小正常  (liver / normal in size and shape) | 574 | 52.71% | 6.99% |
| 肝脏 / 轮廓规整  (liver / contour is regular) | 521 | 47.84% | 6.35% |
| 肝门 / 未见异常  (porta hepatis / regular) | 513 | 47.10% | 6.25% |
| 肝裂 / 无增宽  (hepatic fissures / no broadening) | 432 | 39.77% | 5.26% |
| 肝叶 / 比例如常  (liver lobe / normal proportion) | 427 | 39.21% | 5.20% |
| 肝脏 / 低密度影  (liver / low density) | 302 | 27.73% | 3.68% |
